# Germline homozygous missense *DEPDC5* variants cause severe refractory early-onset epilepsy, macrocephaly and bilateral polymicrogyria

**DOI:** 10.1093/hmg/ddac225

**Published:** 2022-09-06

**Authors:** Athina Ververi, Sara Zagaglia, Lara Menzies, Julia Baptista, Richard Caswell, Stephanie Baulac, Sian Ellard, Sally Lynch, Genomics England Research Consortium, Thomas S Jacques, Maninder Singh Chawla, Martin Heier, Mari Ann Kulseth, Inger-Lise Mero, Anne Katrine Våtevik, Ichraf Kraoua, Hanene Ben Rhouma, Thouraya Ben Younes, Zouhour Miladi, Ilhem Ben Youssef Turki, Wendy D Jones, Emma Clement, Christin Eltze, Kshitij Mankad, Ashirwad Merve, Jennifer Parker, Bethan Hoskins, Ronit Pressler, Sniya Sudhakar, Catherine DeVile, Tessa Homfray, Marios Kaliakatsos, Ponnudas (Prab) Prabhakar, Robert Robinson, Sara Margrete Bøen Keim, Imen Habibi, Alexandre Reymond, Sanjay M Sisodiya, Jane A Hurst

**Affiliations:** Department of Clinical Genetics & Genomic Medicine, Great Ormond Street Hospital for Children NHS Foundation Trust, London, UK; Genetic Unit, 1^st^ Obstetrics-Gynaecology Department, Aristotle University of Thessaloniki, Papageorgiou General Hospital, Thessaloniki, Greece; Department of Clinical and Experimental Epilepsy, UCL Queen Square Institute of Neurology, London, UK; Chalfont Centre for Epilepsy, Chalfont St. Peter, UK; Department of Clinical Genetics & Genomic Medicine, Great Ormond Street Hospital for Children NHS Foundation Trust, London, UK; Faculty of Health, University of Plymouth, Plymouth, UK; Exeter Genomics Laboratory, Royal Devon University Healthcare NHS Foundation Trust, Exeter, UK; Institut du Cerveau - Paris Brain Institute - ICM, Inserm, CNRS, Sorbonne Université, F-75013 Paris, France; Exeter Genomics Laboratory, Royal Devon University Healthcare NHS Foundation Trust, Exeter, UK; Academic Centre on Rare Diseases, University College Dublin School of Medicine and Medical Science, Dublin, Ireland; Department of Clinical Genetics, Children's Health Ireland (CHI) at Crumlin, Dublin, Ireland; Developmental Biology and Cancer Research and Teaching Department, UCL Great Ormond Street Institute of Child Health, London, UK; Department of Histopathology, Great Ormond Street Hospital for Children NHS Foundation Trust, London, UK; Department of Neuroradiology, Oslo University Hospital, Oslo, Norway; Department of Clinical Neuroscience for Children, Oslo University Hospital, Oslo, Norway; Department of Medical Genetics, Oslo University Hospital, Oslo, Norway; Department of Medical Genetics, Oslo University Hospital, Oslo, Norway; National Center for Epilepsy-SSE, Oslo Univeristy Hospital, Oslo, Norway; Research Laboratory LR18SP04, Department of Child and Adolescent Neurology, National Institute Mongi Ben Hmida of Neurology, Tunis, Tunisia. Faculty of Medicine of Tunis, University of Tunis El Manar, Tunis, Tunisia; Research Laboratory LR18SP04, Department of Child and Adolescent Neurology, National Institute Mongi Ben Hmida of Neurology, Tunis, Tunisia. Faculty of Medicine of Tunis, University of Tunis El Manar, Tunis, Tunisia; Research Laboratory LR18SP04, Department of Child and Adolescent Neurology, National Institute Mongi Ben Hmida of Neurology, Tunis, Tunisia. Faculty of Medicine of Tunis, University of Tunis El Manar, Tunis, Tunisia; Research Laboratory LR18SP04, Department of Child and Adolescent Neurology, National Institute Mongi Ben Hmida of Neurology, Tunis, Tunisia. Faculty of Medicine of Tunis, University of Tunis El Manar, Tunis, Tunisia; Research Laboratory LR18SP04, Department of Child and Adolescent Neurology, National Institute Mongi Ben Hmida of Neurology, Tunis, Tunisia. Faculty of Medicine of Tunis, University of Tunis El Manar, Tunis, Tunisia; Department of Clinical Genetics & Genomic Medicine, Great Ormond Street Hospital for Children NHS Foundation Trust, London, UK; Department of Clinical Genetics & Genomic Medicine, Great Ormond Street Hospital for Children NHS Foundation Trust, London, UK; Department of Paediatric Neurology, Great Ormond Street Hospital for Children NHS Foundation Trust, London, UK; Department of Radiology, Great Ormond Street Hospital for Children NHS Foundation Trust, London, UK; Department of Histopathology, Great Ormond Street Hospital for Children NHS Foundation Trust, London, UK; North Thames Genomic Laboratory Hub, Great Ormond Street Hospital for Children NHS Foundation Trust, London, UK; North Thames Genomic Laboratory Hub, Great Ormond Street Hospital for Children NHS Foundation Trust, London, UK; Department of Clinical Neurophysiology, Great Ormond Street Hospital for Children NHS Foundation Trust, London, UK; Department of Radiology, Great Ormond Street Hospital for Children NHS Foundation Trust, London, UK; Department of Paediatric Neurology, Great Ormond Street Hospital for Children NHS Foundation Trust, London, UK; SW Thames Regional Genetics Service, St George's Hospital, St George's University of London, London, UK; Department of Paediatric Neurology, Great Ormond Street Hospital for Children NHS Foundation Trust, London, UK; Department of Paediatric Neurology, Great Ormond Street Hospital for Children NHS Foundation Trust, London, UK; Department of Paediatric Neurology, Great Ormond Street Hospital for Children NHS Foundation Trust, London, UK; Department of Medical Genetics, Oslo University Hospital, Oslo, Norway; Center for Integrative Genomics, University of Lausanne, Lausanne, Switzerland; Center for Integrative Genomics, University of Lausanne, Lausanne, Switzerland; Department of Clinical and Experimental Epilepsy, UCL Queen Square Institute of Neurology, London, UK; Chalfont Centre for Epilepsy, Chalfont St. Peter, UK; Department of Clinical Genetics & Genomic Medicine, Great Ormond Street Hospital for Children NHS Foundation Trust, London, UK

## Abstract

*DEPDC5* (DEP Domain-Containing Protein 5) encodes an inhibitory component of the mammalian target of rapamycin (mTOR) pathway and is commonly implicated in sporadic and familial focal epilepsies, both non-lesional and in association with focal cortical dysplasia. Germline pathogenic variants are typically heterozygous and inactivating. We describe a novel phenotype caused by germline biallelic missense variants in *DEPDC5*. Cases were identified clinically. Available records, including magnetic resonance imaging and electroencephalography, were reviewed. Genetic testing was performed by whole exome and whole-genome sequencing and cascade screening. In addition, immunohistochemistry was performed on skin biopsy. The phenotype was identified in nine children, eight of which are described in detail herein. Six of the children were of Irish Traveller, two of Tunisian and one of Lebanese origin. The Irish Traveller children shared the same *DEPDC5* germline homozygous missense variant (p.Thr337Arg), whereas the Lebanese and Tunisian children shared a different germline homozygous variant (p.Arg806Cys). Consistent phenotypic features included extensive bilateral polymicrogyria, congenital macrocephaly and early-onset refractory epilepsy, in keeping with other mTOR-opathies. Eye and cardiac involvement and severe neutropenia were also observed in one or more patients. Five of the children died in infancy or childhood; the other four are currently aged between 5 months and 6 years. Skin biopsy immunohistochemistry was supportive of hyperactivation of the mTOR pathway. The clinical, histopathological and genetic evidence supports a causal role for the homozygous *DEPDC5* variants, expanding our understanding of the biology of this gene.

## Introduction

The mammalian target of rapamycin (mTOR) pathway is central to many aspects of intracellular function, including the regulation of cellular growth and cell proliferation (1). In the brain, the pathway is active from the early stages of development and is implicated in neuronal differentiation and growth, synaptogenesis and dendrite formation, thus playing a key role in shaping the hexalaminar cytoarchitecture of the cerebral cortex (2).

The mTOR pathway is subject to strict regulation. *DEPDC5* (DEP Domain-Containing Protein 5) encodes a GTP-ase which, together with NPRL2 and NPRL3 (Nitrogen Permease Regulator-like 2 and 3, respectively), constitutes the GATOR-1 (gap activity towards RAGS-1) complex, an inhibitor of the mTOR pathway. Mutations in *DEPDC5* cause a broad spectrum of focal epilepsies, both non-lesional (3,4) and associated with focal cortical dysplasia (FCD) Type II (5–7). *DEPDC5* mutations were initially found in familial epilepsies with autosomal dominant inheritance (3–5). It has since become clear that *DEPDC5* is one of the most commonly implicated genes in both sporadic and familial focal epilepsies (8,9).

Disease-causing germline variants described so far in *DEPDC5* are heterozygous and in most cases inactivating, including nonsense, frameshift and splice-site variants (10). The most strongly established pathogenic mechanism is haploinsufficiency, with loss of the inhibitory function of the GATOR-1 complex causing hyperactivation of the mTOR pathway (7,11,12), reflected by the histopathological characteristics of FCD Type II (i.e. disrupted cortical lamination and cytomegalic features).

The pathogenicity of heterozygous missense *DEPDC5* variants is less clear due to limited functional evidence (13). Recently, an algorithm for pathogenicity, specific for missense variants in the GATOR-1 genes, was proposed, based on allele frequencies and *in silico* prediction tools (10).

A two-hit model has been suggested to explain phenotypic gradients, with evidence of a second, somatic, loss-of-function variant present within brain regions involved in FCD, on a background of a germline heterozygous inactivating variant^6^. Compound heterozygous variants (germline plus somatic) in *DEPDC5* have been reported, supporting this model (5,11,14–16).

Here, we report two germline recessive homozygous missense variants in *DEPDC5*, identified in nine children from five families with a characteristic severe neurological phenotype and some with additional systemic features. The affected individuals were of Irish Traveller, Lebanese and Tunisian origin. The Irish Travellers are a small nomadic population operating a clan-like structure (17). Such family structuring results in high frequency and familial occurrence of autosomal recessive disorders caused by homozygous variants, due to the presence of founder mutations (18). Both the Lebanese and Tunisian families also showed consanguinity.

## Results

### Clinical phenotypes

**Figure 1:**
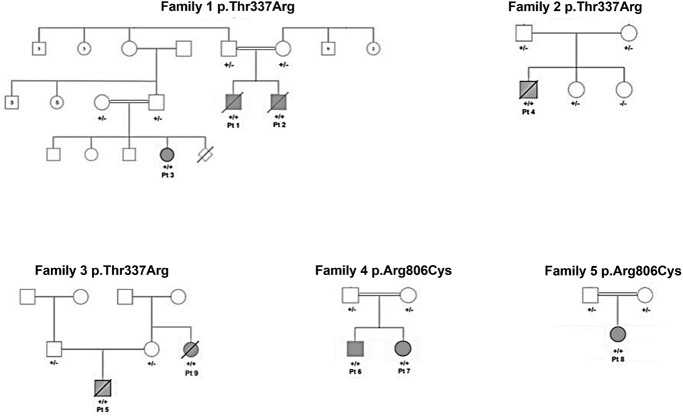
Family trees for Families 1–5.

Family trees are shown in Figure 1. The patients’ phenotypes can be found in Table 1. The second-degree relative of Patient 5 (9th patient) had an identical phenotype and underwent genetic testing. It was not possible to obtain consent to describe the ninth patient in detail.

Further details on the eight patients’ phenotypes can be found in Supplementary Material, S1.

### Neuroimaging

**Figure 2:**
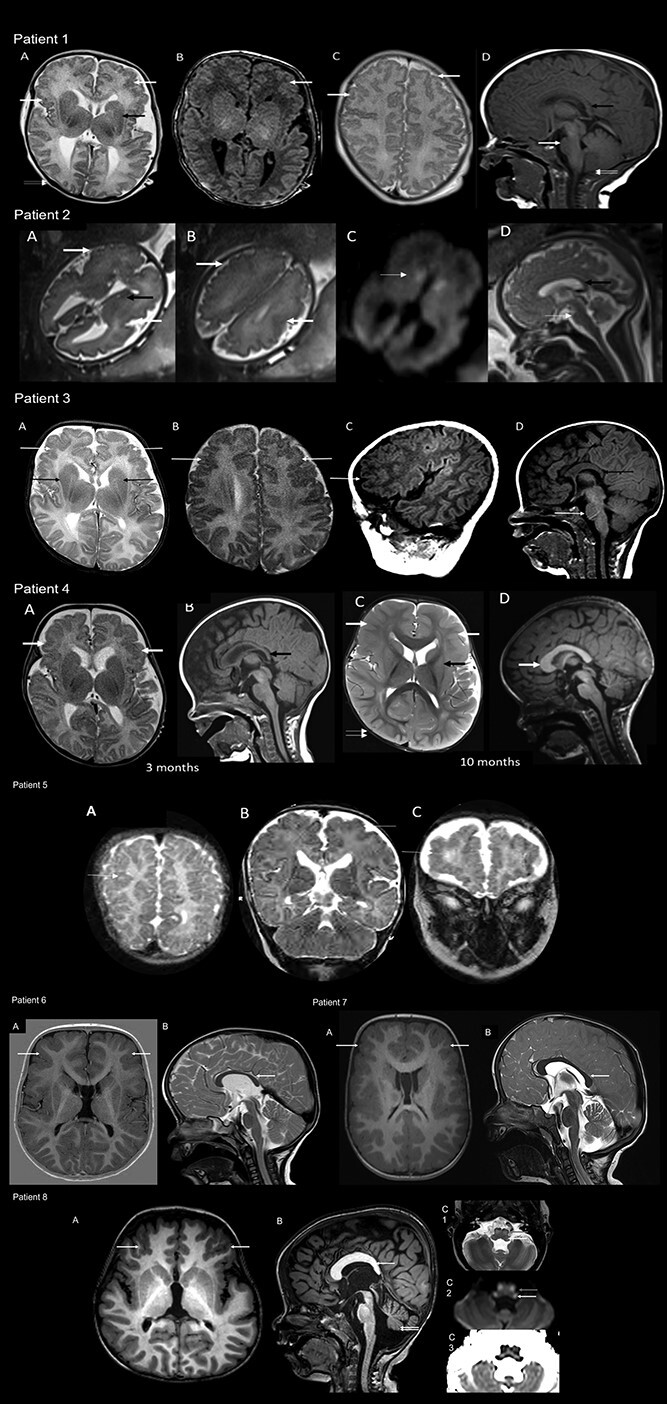
MRI brain imaging from eight affected individuals. Patient 1: MRI at 16 days. T1 and T2 axial images (A–C) showing extensive bilateral frontal and perisylvian polymicrogyria and dysgyria (white arrows) with dysmorphic basal ganglia (black arrow in A). Right occipital plagiocephaly is also seen (double arrows). Midline T1 sagittal image (C) shows thin and posteriorly drooping morphology of the corpus callosum (black arrow), hypoplastic pons (white arrow), as well as inferiorly pointed morphology of the cerebellar tonsil (double arrows). Macrocephaly and frontal bossing with large volume of frontal lobes are also apparent. Patient 2: Fetal MRI at 30 + 3 weeks of gestation. T2 axial images (A, B) showing bilateral polymicrogyria with predominant anterior involvement (white arrows). Cystic changes of the ganglionic eminence are noted (black arrow). There is corresponding restricted diffusion in DWI images (C) shown by the white arrow. The biparietal diameter and head circumference (measurements not shown) correspond to 35 weeks, suggestive of macrocephaly. Midline T2 sagittal image (D) shows a small volume pons (white arrow). The corpus callosum is fully formed (black arrow). The frontal lobes are relatively large in size. Patient 3: MRI at 7w 5d. T2 axial images (A, B) and T1 parasagittal image(C) showing extensive bilateral frontal and polymicrogyria like cortex and dysgyria (white arrows) with dysmorphic basal ganglia (black arrow in A). Midline T1 sagittal image (D) shows thin and posteriorly drooping morphology of the corpus callosum (black arrow) and hypoplastic pons (white arrow). Patient 4: MRIs at 3 months and repeated at 10 months. T2 axial image and midline T1 sagittal image (A, B) at 3 months show macrocephaly with frontal and anterior perisylvian polymicrogyria (white arrows in A). Mild posterior drooping morphology of the corpus callosum is seen (black arrow in B). T2 axial image and midline T1 sagittal image at 10 months (C, D) show large frontal lobes with bilateral extensive anterior predominant polymicrogyria (white arrows in C). The basal ganglia appear dysmorphic (black arrow in C). The corpus callosum shows additional anterior thickening (white arrow in D). Right occipital plagiocephaly is seen (double arrows C). Patient 5: Available MRI images were limited and of reduced quality however T2 images (A, B, C) indicate extensive polymicrogyria-like cortex with predominant fronto-parietal distribution (white arrows). Patients 6 and 7: Show macrocephaly with frontal bossing and squared appearance of frontal bone. Dysmorphic callosum and basal ganglia are evident. Small pontine volume can also be appreciated. Patient 8: Also shows bifrontal polymicrogyria and dysmorphic callosum with anterior thickening and posterior underdevelopment as well as vertical morphology. Small volume pons and cerebellar vermis (double arrows) are appreciable. Panel C shows T2, DWI and ADC (1–3) signal changes suggestive of diffusion restriction in inferior olivary nuclei and inferior cerebellar peduncles.

Bilateral polymicrogyria and macrocephaly were common shared features in the cohort. Abnormalities of the corpus callosum, pons and basal ganglia were also evident. Magnetic resonance imaging (MRI) findings are illustrated in Figure 2.

### Electroencephalography findings

**Figure 3:**
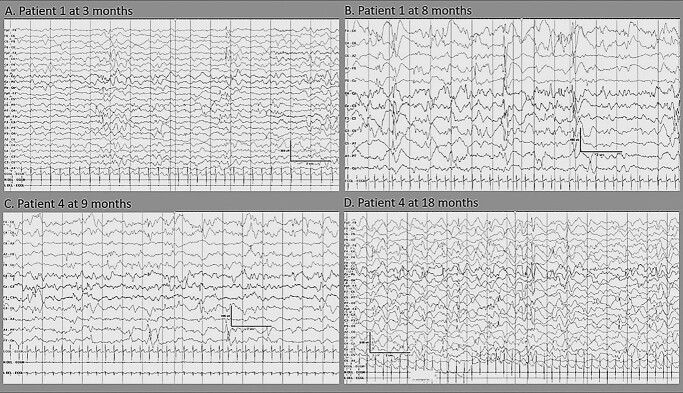
Interictal EEG features of two patients with the homozygous *DEPDC5* variant indicating progressive epileptic encephalopathy. Top row: Patient 1: (**A**) at 3 months, there is only mild excess of intermittent slow, with multifocal sharp waves (left > right) and (**B**) at 8 months, there is marked slowing with frequent multifocal discharges. Bottom row: Patient 4: (**C**) at 9 months the background activity is slow with multifocal discharges independently over both hemispheres and (**D**) at 18 months, the background activity shows a marked excess of slow, absence of age-appropriate sleep phenomena and multifocal sharp and slow wave and spikes.

**Figure 4:**
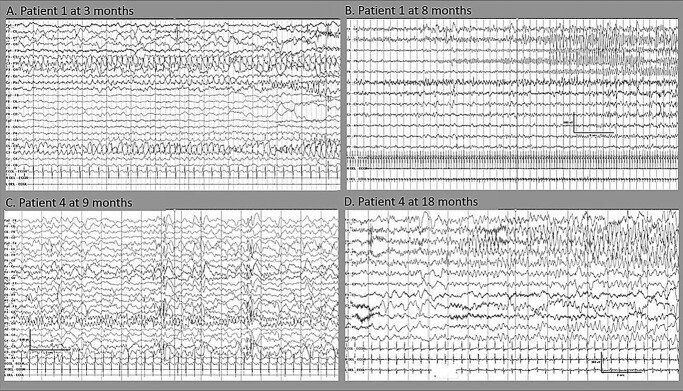
Ictal EEG features of two patients with *DEPDC5* indicating progressive epileptic encephalopathy. Top row: Patient 1 with frequent independent focal seizures during the first year of life: (**A**) subtle seizure characterized by eye deviation to the left with onset from the left parietal region at the age of 3 months and (**B**) frequent multifocal electrographic seizures at the age of 8 months which mostly had an onset from the right central region (B) or less often, from the left parietal region (not shown). Bottom row: Patient 4 with frequent independent focal seizures from either hemisphere at the age of nine and 18 months: (**C**) clinical seizures characterized by eye deviation to the right and left hypotonia with onset from right temporal and (**D**) clinical seizures characterized by subtle tonic posturing of the right hand followed by clonic movements of his right leg with onset from left posterior quadrant.

Longitudinal electroencephalographies (EEGs) displayed features of progressive epileptic encephalopathy with increasingly frequent multifocal discharges of variable side emphasis (Fig. 3). Frequent, often long, clinical and electrographic seizures with multifocal onset from either hemisphere were evident (Fig. 4). Seizures were often electrographic only or characterized by subtle features of eye deviation and autonomic disturbance.

### Genetic analysis

All eight patients had normal array CGH or SNP array.

Patients 1–5 carried the homozygous missense *DEPDC5* variant [NM_ 001242896.3: c.1010C > G, p.(Thr337Arg)]. The second-degree relative of Patient 5 (ninth patient) was also found to carry this homozygous variant.

Patients 6–8 carried the homozygous missense *DEPDC5* variant [NM_001242896.3: c.2416C > T, p.(Arg806Cys)].

The parents of each child were heterozygous for the variant. Family segregation analysis showed that one of the unaffected siblings of Patient 4 was heterozygous for the p.(Thr337Arg) variant and the other sibling did not carry it.

Various further analyses, such as data interrogation for shared rare variants, did not identify other variants of interest in Patients 1 and 3–8 who underwent whole-genome sequencing (WGS) or whole-exome sequencing (WES) (see Supplementary Material, Table S1 and Supplementary Material, S2). Specific interrogation of the WES data of Patients 4 and 5 did not identify other rare variants shared by the two boys. Similarly, interrogation of the WES data of Patients 6 and 7 (siblings) did not identify other rare shared variants with a plausible relationship to the siblings’ phenotype (see Supplementary Material, Table S2).

Additionally, the SNP array data from Patients 1, 2, 3 and 4 were screened for regions of homozygosity (ROH) sized >3 MB. The only shared ROH was located on chromosome 22q12 and contained six OMIM morbid genes, of which only *DEPDC5* was relevant to the patients’ shared phenotype (see Supplementary Material,Table S3).

### Protein modelling

Variants were analysed in structures of GATOR-1 bound to Rag GTPases RagA/RagC and/or the Ragulator complex, in three different states of activation (PDB 7t3a, 7t3b and 7t3c) (19). The overall structure of DEPDC5 was essentially identical in all states, and figures are shown for analysis in PDB 7t3a (inhibited state) only.

**Figure 5:**
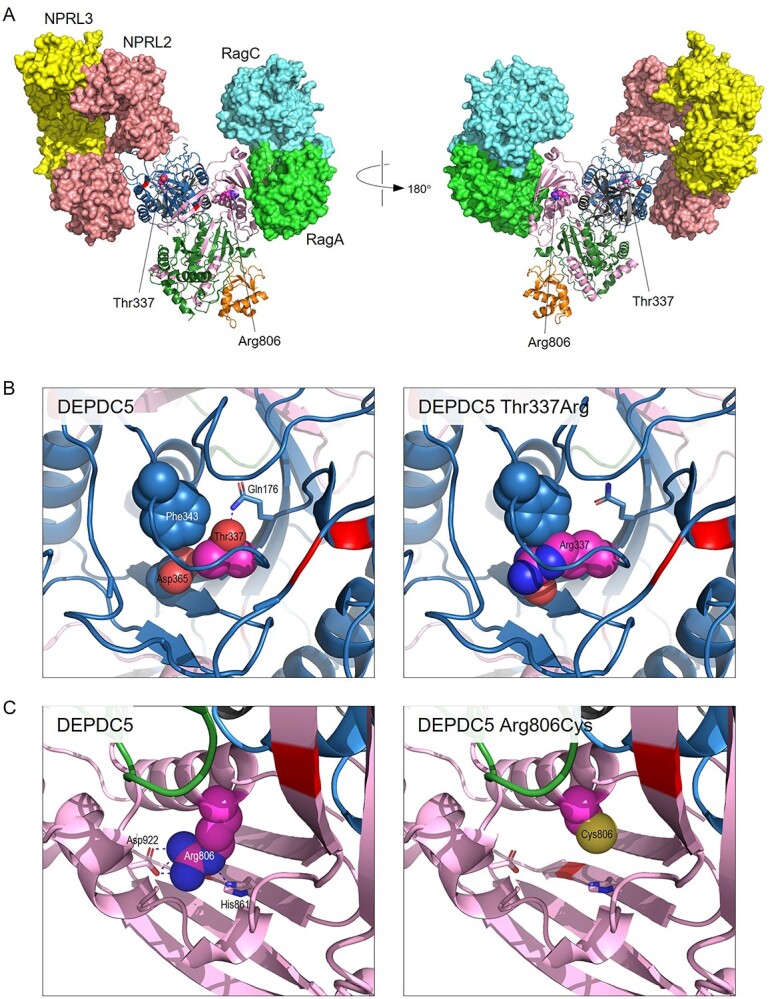
Predicted effect of p.Thr337Arg and p.Arg806Cys variants. (**A**) Structure of the heterotrimeric GATOR1 complex bound to Ran GTPases in the inhibitory state (PDB 7t3a). DEPDC5 is shown in ribbon format, coloured by domain: N-terminal domain (residues 38–165), dark grey; SABA domain (166–425), blue; SHEN domain (721–1010), pink; DEP domain (1175–1270), orange; C-terminal domain (1271–1600), dark green; residues Thr337 and Arg806 are coloured magenta with sidechain atoms shown as space-filling spheres; sites of previously reported pathogenic missense variants (HGMD class DM) are coloured red. Other proteins of the GATOR1 complex (NPRL2, pink; NPRL3, yellow) and Rag GTPases A (green) and C (cyan) are shown with predicted surfaces. (**B**) The left panel shows detail around Thr337 in PDB 7t3a chain A; sidechains atoms of Thr337, Phe343 and Asp365 are shown as space-filling spheres, with carbon atoms coloured as backbone and other atoms by type (red, oxygen); the Gln176 sidechain is shown in stick format, with the broken blue line indicating the hydrogen bond between Thr337 and Gln176 sidechains. The right panel shows the same view of the predicted structure of the p.Thr337Arg variant; the variant was predicted to be severely destabilizing (ΔΔ*G* = 6.39 kcal/mol in PDB 7t3a), primarily due to steric clashes, introduction of a buried charge and loss of hydrogen bonding. (**C**) As B, but showing detail around Arg806 (left) and the p.Arg806Cys variant (right); in the left panel, the broken blue lines show hydrogen bonds from the Arg806 sidechain to those of His861 and Asp962. The p.Arg806Cys variant was also predicted to be severely destabilizing in 7t3a (ΔΔ*G* = 5.21 kcal/mol), due to loss of hydrogen bonding, electrostatic and non-bonded interactions and unfavourable changes in polarity.

The majority of previously reported pathogenic missense variants in *DEPDC5* lie at protein interfaces, either between DEPDC5 and NPRL2 or RagA, or at inter-domain interfaces within DEPDC5 itself (Fig. 5A). In contrast, both Thr337 and Arg806 are completely buried in the DEPDC5 structure; Thr337 lies within the SABA (structural axis for binding arrangement) domain, just below the interface with NPRL2, while Arg806 forms part of the SHEN (steric hindrance for enhancement of nucleotidase activity) domain and lies close to the RagA binding surface.

In the native structure, the Thr337 sidechain lies in close juxtaposition to those of Phe343 and Asp365, while also forming a hydrogen bond to the sidechain of Gln176 (Fig. 5B). This bond was lost in the p.Thr337Arg variant, while the larger sidechain of arginine was predicted to cause steric clashes with those of its near neighbours. The thermodynamic impact of the substitution was calculated using FoldX, which provides a value for *ΔΔG*, the change in free energy of the variant structure compared with that of the native sequence, where values >3 kcal/mol are generally regarded as severely destabilizing (20,21). In PDB 7t3a, the calculated *ΔΔG* value for p.Thr337Arg was 6.39 kcal/mol, while in 7t3b and 7t3c the values were 11.07 kcal/mol and 8.06 kcal/mol respectively, indicating that the variant is likely to result in a loss of stability and a reduced level of functional DEPDC5 protein. Consistent with this, analysis of the p.Thr337Arg variant using Missense3D predicted the variant to cause structural damage as a result of introduction of a buried charged group and breakage of a buried hydrogen bond.

Arg806 lies just below the interface with RagA, and in the native structure provides a link between this interface and the DEPDC5 core by forming hydrogen bonds to the sidechains of His861 and Asp922 (Fig. 5C). These bonds are lost in the p.Arg806Cys variant, while there will also be a loss of non-bonded contacts in the protein core due to the smaller sidechain size of cysteine compared with arginine. Consistent with this, FoldX calculated *ΔΔG* to be 5.21 kcal/mol in PDB 7t3a, with values of 4.36 and 3.30 kcal/mol in 7t3b and 7t3c, respectively, indicating that this variant is also likely to result in reduced protein stability. Missense3D predicted the p.Arg806Cys variant to be structurally damaging due to replacement of a buried charge and breakage of a buried salt bridge (between Arg806 and Asp922).

**Figure 6:**
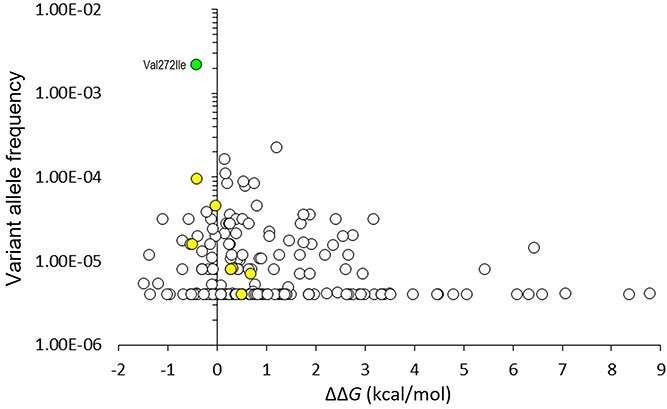
Thermodynamic impact of gnomAD missense variants on DEPDC5 structure. FoldX was used to calculate the thermodynamic impact of all missense variants in the SABA and SHEN domains as reported in gnomAD v2.1.1 and resolved in PDBs entries 7t3a, 7t3b and 7t3c (*n* = 185); the average *ΔΔG* value for each variant was plotted against gnomAD variant allele frequency; the yellow fill shows seven variants which have also been reported in HGMD in association with disease; the green fill shows p.Val272Ile, the only missense variant in structured regions of the SABA or SHEN domains which has been observed as a homozygote in gnomAD; the accepted thresholds for the thermodynamic impact of variants on protein structure are: >3 kcal/mol, severely destabilizing; 1–3 kcal/mol, destabilizing; <1 kcal/mol, neutral or benign^32,33^. Neither p.Thr337Arg nor p.Arg806Cys has been observed in gnomAD; for comparison, these variants yielded average ΔΔ*G* values of 8.51 kcal/mol and 4.269 kcal/mol, respectively.

To examine whether *ΔΔG* values have biological relevance in DEPDC5, *in silico* mutagenesis was used to introduce all variants observed in the gnomAD database within the SABA (structural axis for binding arrangement) and SHEN (steric hindrance for enhancement of nucleotidase activity) domains (residues 166–425, and 721–1010, respectively) structures of DEPDC5 from 7t3a, 7t3b and 7t3c. The total number of variants analysed was 185, which included seven variants which have also been reported in association with disease in the Human Gene Mutation Database (HGMD; http://www.hgmd.cf.ac.uk) (four as class DM, pathogenic, and three as class DM?, possibly pathogenic), and p.Val272Ile, the only variant in the SABA or SHEN domains which has been observed as a homozygote in gnomAD. The average *ΔΔG* value for each variant was plotted against variant allele frequency; this strongly suggests that there is selection against alleles which are structurally damaging, while there is tolerance of those which are neutral or benign, and notably, p.Val272Ile, the only gnomAD variant observed in the homozygous state, was predicted to have no significant impact on DEPDC5 stability (ΔΔ*G* = −0.44 kcal/mol) (Fig. 6). As a group, the average ΔΔ*G* value for all gnomAD missense variants was 1.08 kcal/mol (standard deviation, 1.76 kcal/mol; median, 0.50 kcal/mol). By comparison, the two missense variants reported here were predicted to be substantially more destabilizing, and this difference was highly significant (*P* < 0.0001, Student t-test).

**Table 1:** Phenotypic features of Patients 1–8

	Patient 1	Patient 2	Patient 3	Patient 4	Patient 5	Patient 6	Patient 7	Patient 8
Family	Family 1	Family 1	Family 1	Family 2	Family 3	Family 4	Family 4	Family 5
Origin	Irish Traveller	Irish Traveller	Irish Traveller	Irish Traveller	Irish Traveller	Tunisian	Tunisian	Lebanese
*DEPDC5* variant (NM_001242896.3)	c.1010C > G, p.(Thr337Arg)	c.1010C > G, p.(Thr337Arg)	c.1010C > G, p.(Thr337Arg)	c.1010C > G, p.(Thr337Arg)	c.1010C > G, p.(Thr337Arg)	c.2416C > T, p.(Arg806Cys)	c.2416C > T, p.(Arg806Cys)	c.2416C > T, p.(Arg806Cys)
Consanguinity of parents	2^nd^ cousin parents	2^nd^ cousin parents	2^nd^ cousin parents	Not known	Not known	1^st^ cousin parents	1^st^ cousin parents	1^st^ cousin parents
Sex	Male	Male	Female	Male	Male	Male	Female	Female
Age at inclusion	Died aged 37 months due to respiratory infection	Died aged 1 day due to congenital cardiac disease	10 months old	Died aged 22 months due to recurrent apnoeas	Died aged 15 months due to seizure-related respiratory arrest	6 years old	4 years old	3.5 years old
Antenatal concerns	Antenatal scans not routinely performed	Hypoplastic left heart on fetal ultrasound scan, polymicrogyria on fetal MRI at 30^+3^ weeks	Increased nuchal fold and echogenic bowel on 1^st^ fetal ultrasound scan at 23 weeks	Ventriculomegaly and macrocephaly on fetal ultrasound scan, IUGR suspected	No abnormalities noted on fetal ultrasound scan. Severe pre-eclampsia and pathological CTG at 28 weeks	None recorded	None recorded	None recorded
Birth details	GA: 35 weeks. Emergency CS for maternal preeclampsia	GA: 32 weeks. Emergency CS for maternal preeclampsia	GA: 35 weeks. Vaginal delivery	GA: 36 + 1 weeks. CS due to IUGR and breech presentation.	GA: 28 + 4 weeks. Emergency CS due to abnormal CTG	GA: 35 weeks. Vaginal delivery	Born at term, IUGR	GA: 35 + 5 weeks. Emergency CS for maternal preeclampsia
Neonatal concerns	None recorded	Died on day 1	Admitted to SCBU for feeding difficulties.	Admitted to SCBU for feeding difficulties.	Admitted to NICU, required respiratory support (intubation, CPAP, nasal oxygen) for 90 days	None recorded	None recorded	Hypotonia, feeding difficulties
Birthweight centile (for corrected age)	>99^th^	83^rd^	70^th^	13^th^	13^th^	33^rd^	1^st^	10^th^
Birth length centile (for corrected age)	>99^th^	Not available	Not available	Not available	Not available	89^th^	Not available	3^rd^
Birth head circumference centile (for corrected age)	>99.6^th^	Macrocephaly noted antenatally		84^th^		91^st^	45^th^	>97^th^
Growth parameters centiles for corrected age at last examination	At 6 months: Wt > 99.6^th^, HC > 99.6^th^	Not applicable	At 5 months: Wt 9^th,^ Lt 15^th^, HC 50^th^	At 7 months: Wt < 0.4th, Lt < 0.4^th^, HC > 99.6^th^	At 7 months: Wt 4^th^, Lt 1^st^, HC 98^th^	At 4 years: HC > 99.6^th^	At 16 months: Wt: 56^th^ Lt: 18th HC > 99.6^th^	Macrocephaly
Notable physical features	Macrocephaly with frontal bossing, capillary malformation sacral area	Not applicable	Frontal bossing	Macrocephaly with frontal bossing, wide open fontanelle, bilateral single palmar creases, capillary malformation back of neck	Macrocephaly with frontal bossing, hypertelorism, midface hypoplasia	Macrocephaly with frontal bossing	None recorded	Macrocephaly with frontal bossing, bushy eyebrows and long eyelashes, low set ears and thin, curly hair.
Epilepsy	Multifocal-onset seizures at 4 weeks. Seizures refractory to multiple antiepileptic medications and to ketogenic diet	Not applicable	Multifocal-onset seizures at 3 weeks. Refractory to multiple antiepileptic medications. At 5 months clusters of seizures and episodes of desaturation	Convulsive status epilepticus lasting 2 h at 8 months, followed by permanent left hemiparesis. Subsequently, focal-onset seizures, increasingly prolonged and frequent over time. Trials of multiple medications and classical ketogenic diet ineffective.	Infantile spasms, commenced on prednisolone. Subsequently, multifocal onset seizures, refractory to multiple antiepileptic medications. Prolonged episodes of apnoea and recurrent episodes of aspiration.	Generalized tonic–clonic? seizures since 14 months. Currently focal and generalized seizures, relapsing frequently and necessitating adjustment of medication. Currently on valproate	Febrile focal seizures since 3 years, currently on valproate	Generalized tonic–clonic seizures at 9 months, initially well controlled with levetiracetam, and later lacosamide. At 15 months hospitalized due to increasing seizures, which over 1 week evolved to convulsive status epilepticus. Currenty seizure free on ketogenic diet in combination with topiramate
Psychomotor development	Could sit momentarily and smile socially until 13 months, when skills were lost. No further psychomotor development until the end of life (37 months)	Not applicable	5 months: smiling but not fixing or following. Poor head control	Unable to sit independently and was non-verbal until the end of life (22 months). Could fix and follow, but inconsistently. Orally fed except at times of increased seizure activity, when tube feeding was required	Unable to fix and follow, did not sit and was non-verbal until the end of life (15 months).	Held his at 8 months, sat at 18 months, babbled at 3 years 6 years: not standing, no ambutation and no speech	Held head at 5 months, sat unaided at 10 months and stood at 2 years. 4 years: no ambutation and no speech	3.5 years: not rolling over or sitting. No ambutation and no speech
Visual system abnormalities	Electrodiagnostic evidence of marked pan-retinal rod-cone dystrophy, severely sight-impaired	Not applicable	No. Electrophysiology: normal retinal function, some VEP evidence of activation of visual pathways but poor quality due to presumed EEG activity and lack of attention.	No. Bilateral healthy disc and macula, no signs of retinal dystrophy. Slightly pale fundus.	Normal initially, aged 7 m very hypopigmented retina with grey macula noted	Poor ocular tracking, amblyopia	Poor ocular tracking, horizontal nystagmus	None recorded
Other systemic abnormalities	Feeding difficulties, requiring PEG-tube feeding Constitutionally small right kidney (normal overall renal function)	Hypoplastic left heart syndrome	Left pulmonary artery stenosis	Sinus tachycardia with occasional premature ventricular complexes at 18 months. 24-h Holter recording normal	Renal stone, hypertension, biventricular hypertrophy, cryptorchidism, umbilical hernia	Inguinal hernia, surgically repaired at 3 months	None recorded	Feeding difficulties, requiring PEG-tube feeding After episode of status epilepticus at 15 months: Persistent hyponatremia and hypoosmolality combined with high renin, high aldosterone, low urine sodium output and high urine osmolality. Not responding to treatment with a competitive vasopressin receptor 2 antagonist and requiring high dose sodium supplements. Hyponatremia worsens during febrile episodes.
Immunological concerns	Recurrent respiratory tract infections requiring multiple PICU admissions, neutropenia of unknown cause during admissions. Death secondary to respiratory tract infection	Not applicable	None recorded	Sepsis (multiple pathogens on PCR on blood) and neutropenia of unknown cause during admissions	Treated for possible sepsis, RSV positive during last admission. Mild neutropenia of unknown cause during one of his admissions	None recorded	None recorded	None recorded
MRI findings	Extensive bilateral frontal and perisylvian polymicrogyria and dysgyria with dysmorphic basal ganglia. Thin and posteriorly drooping morphology of the corpus callosum, hypoplastic pons, inferiorly pointed morphology of the cerebellar tonsil. Macrocephaly and frontal bossing with large volume of frontal lobes	Bilateral polymicrogyria with predominant anterior involvement, cystic changes of the ganglionic eminence. Biparietal diameter and head circumference corresponding to 35 weeks, suggestive of macrocephaly. Small volume pons and relatively large in size frontal lobes	Extensive bilateral frontal and polymicrogyria-like cortex and dysgyria. Dysmorphic basal ganglia, thin and posteriorly drooping morphology of the corpus callosum and hypoplastic pons	Macrocephaly with frontal and anterior perisylvian polymicrogyria, mild posterior drooping morphology of the corpus callosum, large frontal lobes, dysmorphic basal ganglia, anterior thickening of the corpus callosum	Extensive polymicrogyria-like cortex with predominant fronto-parietal distribution	Macrocephaly with frontal bossing and squared appearance of frontal bone. Dysmorphic callosum and basal ganglia		Bifrontal polymicrogyria, dysmorphic corpus callosum with anterior thickening and posterior underdevelopment, small volume pons and cerebellar vermis

IUGR: intrauterine growth retardation, CTG: cardiotocography, CS: caesarean section, SCBU: Special Care Baby Unit, NICU: Neonatal Intensive Care Unit, CPAP: continuous positive airway pressure, HC: head circumference, Wt: weight, Lt: length, PICU: Paediatric Intensive Care Unit, PCR: polymerase chain reaction, NA: not applicable

In this context, it is highly likely that structural damage and destabilization caused by the p.Thr337Arg and p.Arg806Cys variants will, in the homozygous state, be sufficient to result in a significant loss of DEPDC5 and GATOR-1 function, consistent with increased mTOR activity. Furthermore, the high values of *ΔΔG* calculated for some rare gnomAD variants suggest that some of these might also be pathogenic if present in the homozygous state or in trans with a second damaging variant.

### Immunohistochemistry

**Figure 7:**
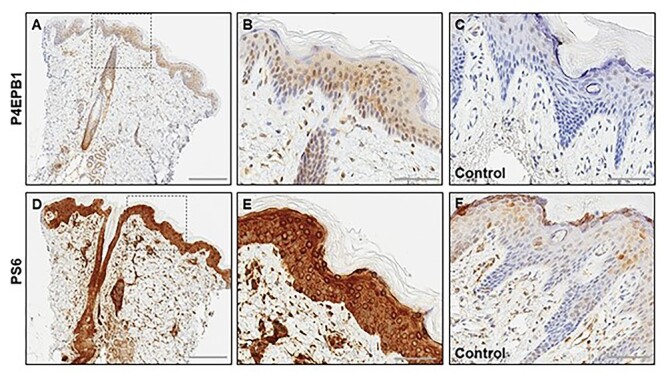
Skin biopsy (from Patient 4) with mTOR effector protein expression: (**A**–**C**) P4EPB1 immunohistochemistry showing expression in the epidermis epithelial cells, adnexal structures and in the dermis (A) with higher magnification of the marked area of the epidermis highlighting expression in many keratinocytes (B), in comparison with the normal control skin (C). (**D**–**F**) PS6 immunohistochemistry showing strong upregulation in the epidermis, hair follicles and the adnexal structures in the dermis (D), with higher magnification of the marked area of the epidermis highlighting diffuse expression in the epidermal cells (E), in comparison with the normal control skin (F). The control tissue in C and F is the same and is a surgical sample of normal skin from a newborn child, not expected to have mTOR overactivity.

Immunohistochemistry for mTOR pathway effectors in the skin sample of Patient 3 showed prominent positive staining for pS6, as well as positive staining for pEBP1 (Fig. 7) compared with control tissue, suggestive of an overall increase in mTOR activity.

### Variant classification

Both variants were absent from the gnomAD population database in heterozygous and homozygous state. The p.Arg806Cys was also absent from the local database of >100 Tunisian individuals of the Center for Integrative Genomics, University of Lausanne. A different amino acid change, p.Thr337Met, in the same position as p.Thr337Arg was found in three heterozygotes, but no homozygotes, in gnomAD.

*In silico* predictive tools (DANN, DEOGEN2, EIGEN, FATHMM-MKL, M-CAP, MutationAssessor, MutationTaster MutationTester, Polyphen-2, Aligh-GVGD, SIFT) unanimously supported a deleterious effect for both variants. The phred CADD scores were 26.7 for the p.Thr337Arg variant and 28.5 for the p.Arg806Cys variant. Protein modelling indicated that both variants have deleterious consequences.

The p.Thr337Arg variant has been reported in heterozygosity by a single submitter in ClinVar as a variant of uncertain significance. We confirmed with the submitter that the test had been requested for a condition other than epilepsy, which the subject did not have. The p.Arg806Cys variant has been reported in heterozygosity by three submitters in ClinVar as a variant of uncertain significance.

Based on evidence from bioinformatics, modelling, consistent clinical features and segregation analysis, the variants were classified as likely pathogenic (class 4) in the homozygous state, using ACMG and ACGS criteria and the framework proposed by Baldassari *et al*. (13). The criteria used, in detail, for the p.Thr337Arg and the p.Arg806Cys included PM3_Moderate (the homozygous variants were identified in a total of six and three patients, respectively, thought to originate from at least two apparently unrelated families with clinical features consistent with *DEPDC5*-related disorder), PM2_Supporting (the variants have not been reported in the gnomAD database), PP1_Supporting (the variants co-segregate with disease in multiple affected family members), PP3_Supporting (the variants are highly conserved, and are predicted by SIFT, PolyPhen and AlignGVGD to have a deleterious effect on protein function) and PP4_Supporting (the patients had clinical features compatible with an mTOR pathway-related epilepsy and a skin biopsy in Patient 4 was supportive of an mTOR pathway disorder).

## Discussion

We describe a characteristic severe neurologic phenotype caused by two homozygous *DEPDC5* missense variants, p.Thr337Arg and p.Arg806Cys, identified in six and three children, respectively. Affected individuals demonstrated extensive bilateral polymicrogyria, early-onset refractory epilepsy and severe developmental delay. Six of the children also exhibited macrocephaly. This novel recessive phenotype differs significantly from the epilepsy (with or without FCD) phenotype previously reported with heterozygous loss-of-function germline *DEPDC5* variants (6–8).

Global developmental delay was a universal feature in our patients. This worsened in severity in Patients 1, 4, 5 and 8 after the onset of seizures. Longitudinal EEGs showed progressive worsening of the encephalopathic features, in conjunction with an increased frequency of multifocal epileptiform discharges and electroclinical seizures. These features are consistent with a phenotype of developmental and epileptic encephalopathy (22).

The children also showed variable systemic extracerebral involvement. While the neurological features were strikingly similar, extracerebral manifestations were less consistent and included eye involvement, severe transient neutropenia, congenital heart defect, constitutionally small kidney and severe hyponatremia and hypo-osmolarity. The presence of multisystemic features is common in other mTORopathies, such as the well-known cardiac, renal and ophthalmological manifestations frequently seen in tuberous sclerosis. However, given the variability of extra-cerebral features in our cohort and the small number of patients so far described, it remains to be seen whether a characteristic extra-neural phenotype emerges that is causally related to *DEPDC5*.

All eight patients described in detail had strikingly similar findings on brain imaging. These features included bilateral polymicrogyria with anterior predominance (frontal, anterior parietal and perisylvian regions) in all patients. The corpus callosum was dysmorphic in several patients with thickened anterior segments and a posterior drooping morphology. In six patients, pontine volume was less than expected. The basal ganglia were dysmorphic in seven patients, with posterior tapering of the caudate. Macrocephaly was present in seven of the eight patients and most patients had increased frontal lobe volume, which became more conspicuous with age. A squared appearance of the frontal bone was also demonstrated in most. Patient 8 showed symmetrical T2 hyper-intensities with corresponding diffusion restriction along the inferior olivary nuclei and inferior cerebellar peduncles, the cause for which remains unknown.

Macrocephaly in the absence of cerebral ventriculomegaly is a key discriminating feature of mTORopathies, having been described as part of the phenotypic spectrum caused by variants in other genes involved in the regulation of the mTOR pathway, including autosomal dominant variants in *PTEN* (23) and biallelic variants in *TBCK* (24), *HERC1* (25), *TBC1D7* (26) and *STRADA* (27). Polymicrogyria is one of the most common malformations of cortical development, characterized by abnormal cortical lamination and excessive folding of the cortical surface (28), and while this is most commonly associated with microcephaly or normal head size, an association with macrocephaly (which was prominent in our cohort) is typically related to mTOR-hyperactivating mutations in genes of the PI3K-AKT-mTOR pathway (29–31).

The skin biopsy immunostaining were consistent with mTOR hyperactivation, providing indirect, albeit non-specific, evidence for a functional impact of the p.Thr337Arg variant at least.

Given the differences in the phenotype observed in our patients compared with that previously reported in *DEPDC5*-associated disease, we made considerable efforts to identify possible candidate genes and variants from patient WES, WGS and SNP array data. The homozygous *DEPDC5* variants p.Thr337Arg and p.Arg806Cys were the only shared variants in our cohort and the only plausible candidates remaining after filtering.

On the basis that pathogenic germline variants in *DEPDC5* are believed to cause disease as a result of haploinsufficiency, it follows that missense variants, which cause a substantial loss of DEPDC5 activity, could also result in disease when present in the homozygous state or in trans with a second deleterious variant or, rarely, on their own, if they have severe enough consequences. Pathogenic missense variants may cause partial or complete loss of function either by disrupting a critical property of the protein (e.g. catalytic activity, ligand binding or protein–protein interaction), or by causing structural destabilization leading a reduced level of functional protein as a result of misfolding, increased degradation or both (20,21).

Both the p.Thr337Arg and p.Arg806Cys variants were predicted to be severely destabilizing at the molecular level, and with a magnitude which was predicted to be significantly higher than that for missense variants reported in gnomAD. However, whereas structural destabilization is a common mechanism of loss-of-function variants, missense variants may also have a deleterious impact by affecting specific functions such as catalysis, ligand binding or protein–protein interactions. Interestingly, of the eight missense variants currently reported in HGMD as pathogenic, five lie at known interfaces, either for interaction of DEPDC5 with NPRL2 or RagA or at inter-domain interfaces within DEPDC5 itself, and of these, only two are predicted to have a significant impact on protein stability (p.Met181Lys, average ΔΔ*G* = 1.96 kcal/mol; and p.His214Asp, average ΔΔ*G* = 2.88 kcal/mol). A further two variants, p.Arg247His, p.Arg997Cys) result in loss of surface charge in a region which could potentially interact with substrates or with other, as yet unknown binding partners. In this context, the p.Thr337Arg and p.Arg806Cys variants reported here are unusual in that they are both predicted to cause severe destabilization at the molecular level. However, based on the absence of phenotype in heterozygous carriers of these variants, we conclude that the two variants result in a partial loss of function, causing disease only in homozygosity, and so given that there appears to be selection against strongly destabilizing variants in the general population (as shown by thermodynamic analysis of gnomAD variants), it is perhaps not surprising that such homozygous variants have not been observed previously. Nevertheless, as genetic analysis becomes more widespread, the identification of novel individual missense variants, or new combinations of rare but potentially damaging variants, is increasingly leading to a broadening of phenotypic spectra, with recessive phenotypes (sometimes of greater severity, or of completely different features) being described for genes previously associated with ‘dominant’ conditions (32).

In addition to the homozygous *DEPDC5* variants in our cohort, another germline homozygous *DEPDC5* variant, p.Pro1031His, was recently identified in a 5-year-old girl with FCD and childhood-onset epilepsy (33). The authors proposed a molecular subregional effect, according to which variants closer to the NPRL2/NPRL3 binding site (where DEPDC5 binds to exert its inhibitory effect on the mTOR pathway) may lead to more severe phenotypes featuring cortical dysplasia (34). However, Pro1031 lies in an unstructured region of the protein which is not resolved in experimental structures, and thus the effect of the Pro1031His variant may be mediated by regulatory rather than structural effects. Interestingly, Ser1028 is reported in the PhosphoSitePlus database (https://www.phosphosite.org) to be phosphorylated, and it is possible that this modification is affected by the Pro1031His variant, although the functional consequences are as yet unknown.

The lack of functional validation of the effect of the two variants on protein stability is a significant limitation of this study. Also, the unusual characteristics of our cohort, with a high degree of relatedness among patients and the broad spectrum of extra-neurological features, were major challenges for the ascertainment of the pathogenicity of the two *DEPDC5* variants. Despite these limitations, the pathogenicity of the two variants is clearly indicated by the combination of evidence presented: the consistent MRI features (well in-keeping with other mTORopathies), the result of the skin biopsy and the findings of the protein modelling. Furthermore*,* given the differences in the phenotype observed in our patients compared with that previously reported in *DEPDC5*-associated disease, we extensively searched for possible candidate genes and variants from patient WES, WGS and SNP array data. The homozygous *DEPDC5* variants p.Thr337Arg and p.Arg806Cys were the only shared variants in our cohort and the only plausible candidates remaining after filtering.

We have, additionally, applied the ClinGen scoring system to quantify the evidence supporting the *DEPDC5*-phenotype association. The curated evidence included case-level data and experimental data as detailed already in the manuscript. Based on the ClinGen Gene-Disease Validity Standard Operating Procedures, Version 9 (35), the available evidence reached the level of moderate. While more evidence is needed to establish this relationship definitively, no convincing contradictory evidence has emerged.

Animal models have been generated to recapitulate pathological changes underlying *DEPDC5*-related epileptogenicity and to better understand gene function. Constitutive *Depdc5* knockout rodents showed severe *in utero* growth delay, micro/anophthalmia, heart defects and embryonic lethality (12). Of further interest, a neuron-specific *Depdc5* conditional knockout mouse model displayed macrocephaly, increased neuron size and dysplastic features consistent with abnormal mTOR activity and seizure susceptibility (36). Acute knockdown of Depdc5 in cultured neurons leads to mTOR hyperactivation, increased cell body size and increased excitatory (but not inhibitory) synaptic transmission and intrinsic excitability. These models provide functional evidence of the pro-epileptogenic effect of *DEPDC5* loss-of-function-related mTOR hyperactivation, and indicate the gradient of phenotype severity described above, in relation to the time of onset and location of DEPDC5 functional loss (12,37). A dose effect is also suggested by the differences between knockdown and knockout models.

Both time of onset and degree of the functional impairment have an impact on tissue distribution of pathology. Thus, hemimegalencephaly and FCD share neuropathological features and common genetic aetiologies: their different spatial extents reflect the occurrence of similar mutations at distinct developmental timepoints (38). A dose-dependent effect was recently highlighted in megalencephaly syndromes associated with pathogenic variants in *PIK3CA*, *PIK3R2*, *AKT* and *MTOR* (39). There is also pre-clinical evidence that the final consequence of hyperactivation of the mTOR pathway depends on the stage of brain development at which it occurs: while in early stages of development in a mouse model with a gain-of-function mTOR mutant, hyperactivation causes neuronal apoptosis and microcephaly, hyperactivation of the mTOR pathway in post-mitotic neurons results in impaired neuronal migration and cellular hypertrophy, with macrocephaly and abnormal cortical architecture (40).

There is thus increasing evidence supporting the hypothesis that, in mTORopathies, the phenotype relates to three axes: dose effect (related to the type and allelic status of the variant); timing of onset of the effect of the mutation (pre-or post-mitotic) and the consequent spatial distribution of tissue alterations. The timing of onset can clearly be determined in model systems; the timing of the occurrence of human brain somatic mutation can only be inferred by the pattern of cortical development.

In the five Irish Traveller children reported here a founder mutation seems likely, proving this is a limitation of our study. The Irish Traveller endogamous population, a nomadic population, operates a clan-like structure. Individuals typically marry within their clan and relationships are frequently consanguineous. Even couples not known to be related may share significant genetic material (18). Lynch *et al*. (18) published SNP-based data showing that the average level of homozygosity in the Irish Traveller population was found to be 8%, compared with 2% in the general Irish population. In our cohort, detailed family trees for the three Irish families did not support relatedness. However, it is often challenging to refine the degree of relatedness in this population, due to issues of privacy, high mobility, reluctance to seek health advice, poor health literacy and, last but not least, lack of consent to examine possible relatedness.

Taken together, the nature of the brain malformations; the absence of other plausible variants; the segregation and the evidence from immunohistology, pathogenicity predictions and *in silico* structural analysis pointed to the conclusion that the homozygous *DEPDC5* variants were causative of the shared phenotype in our cohort. The novel recessive phenotype described here broadens the spectrum of entities associated with *DEPDC5* variants, and, to our knowledge, is the most severe *DEPDC5*-related condition documented so far.

## Materials and Methods

This project was approved by the Great Ormond Street Research and Development and Information Government Offices. Written informed consent for genetic testing, sharing of clinical information and publication was obtained from parents or legal guardians as approved by the relevant institutional review boards.

### Case ascertainment

Patients 1–4 were identified at Great Ormond Street Hospital, London, UK from January to June 2019. Patient 5 was identified through personal communication with the referring clinician from Dublin, Ireland. Patients 6 and 7 were identified at the Center for Integrative Genomics, University of Lausanne, Lausanne, Switzerland. Patient 8 was identified at the Department of Clinical Genetics, Oslo University Hospital, Oslo, Norway. The connection between clinicians in the UK, Switzerland and Norway was made through Genematcher (41).

### Family 1

Patient 1 was referred to the Clinical Genetics service at the age of 4 weeks, due to epilepsy, macrocephaly and polymicrogyria on brain MRI. He had baseline genetic investigations, including a segmental overgrowth panel (*PIK3CA, PTEN, PIK3R2, AKT1, AKT3, CCND2, MTOR*) on genomic DNA extracted from cultured skin fibroblasts. He subsequently underwent trio WGS.

Patient 2 was the younger male sibling of Patient 1. Fetal brain MRI, undertaken because of the family history, showed polymicrogyria. Patient 2 died at day 1 from an inoperable cardiac anomaly. After his death, a homozygous variant in the *DEPDC5* gene was identified in his brother, Patient 1. Subsequently, targeted genetic testing was undertaken on genomic DNA (previously extracted from amniotic fluid) from Patient 2.

Patient 3 is the daughter of a paternal cousin of Patients 1 and 2 (see Fig. 1). She was referred to the Clinical Genetics service at the age of 3 months, due to epilepsy and polymicrogyria on brain MRI. She underwent trio WGS.

### Family 2

Patient 4 was referred to the Clinical Genetics service at the age of 8 months, due to epilepsy, macrocephaly and polymicrogyria on brain MRI. He had normal baseline genetic investigations, including a segmental overgrowth panel (*PIK3CA, PTEN, PIK3R2, AKT1, AKT3, CCND2, MTOR*) on genomic DNA extracted from peripheral blood. He underwent trio WES.

### Family 3

Patient 5 was referred to the Clinical Genetics service at the age of three and a half months due to possible skeletal dysplasia, as he had rhizomelic shortening, and macrocephaly. Robinow syndrome was considered, but testing for *ROR2, WNT5A, DVL1, DVL3* and *NOG* was negative, so he underwent trio WES.

### Family 4

Patient 6 was referred to the Clinical Genetics service at the age of 6 years, due to macrocephaly, epilepsy and developmental delay. He underwent duo WES, along with his affected sister, Patient 7.

Patient 7 was the younger female sibling of Patient 6. She was referred to the Clinical Genetics service, due to macrocephaly, along with the family history. At the age of 4 years, she underwent duo WES, as mentioned above.

### Family 5

Patient 8 was referred to the Clinical Genetics service in the neonatal period, due to hypotonia and macrocephaly. She was re-evaluated due to refractory epilepsy at 15 months, whereupon she underwent trio WES.

### Genome and exome sequencing

WGS in Patient 1 was performed on a research basis through the 100 000 Genomes Project (42). Genomic DNA was extracted from cultured skin fibroblasts of Patient 1 and his parents’ peripheral blood. Sequencing was performed on a HiSeq2500 (Illumina, San Diego, CA, USA) and alignment was performed by Illumina’s Isaac aligner against the reference human genome GRCh37. The length of paired-end reads was 150 bp and the mean depth of coverage was 30×. Clinical genome interpretation was performed using Omicia’s Opal platform (43).

WGS in Patient 3 was performed by the national UK WGS provider (Illumina) and data analysis and interpretation were carried out by the Genomic Laboratory Hub based at Great Ormond Street Hospital. Genomic DNA samples were obtained from peripheral blood of the patient and her parents. The analysis included interrogation of Tier 1 and Tier 2 variants (i.e. variants in ‘green’ genes—confirmed clinically relevant genes). If required, Tier 3 variant analysis was restricted to relevant *de novo* variants and prioritized variants identified by Exomiser (https://www.sanger.ac.uk/tool/exomiser/).

WES in Patients 4 and 5 was performed in the Exeter Genomics Laboratory, UK. Genomic DNA samples were obtained from peripheral blood of the patients and their parents. Whole exome libraries were prepared according to the manufacturer’s instructions using the Agilent SureSelect All Exon capture kit v6 (Santa Clara, USA) or the Twist Core Human Exome protocol (Twist Bioscience, San Francisco, USA). Paired-end short reads were sequenced on a NextSeq 500 (Illumina, San Diego, CA, USA) and alignment was performed by BWA-MEM (v0.7.12) against the reference human genomes GRCh37. A minimum of 60 million reads with >80X mean coverage and >98% of target bases at ≥20X were generated. A bioinformatics pipeline designed by the Exeter Genomics Laboratory was applied to identify rare nonsynonymous variants and variants affecting conserved splice sites or within −50/+10 base pairs of exon-intron boundaries.

Duo WES in Patients 6 and 7 was performed at the Center for Integrative Genomics, University of Lausanne. Genomic DNA of the affected siblings was purified from blood. WES was performed on gDNA of the affected siblings. The exome was captured using the xGen Exome Research Panel v2 (Integrated DNA Technologies) and sequenced using an Illumina HiSeq4000 platform according to the manufacturers’ protocols. The overall mean-depth base coverage was 136- and 125-fold, while on average 93% and 92% of the targeted region was covered at least 20-fold, respectively. Read mapping and variant calling were performed as described in Alfaiz *et al*. (44) and updated in Mattioli *et al*. (45). Briefly, homozygous and heterozygous variants present in both affected siblings in reported ID genes or potential new ID genes with an MAF <1% and <0.1% in the general population (1000genome, EVS, gnomAD), respectively, were retained. Their familial segregation was assessed by Sanger sequencing.

WES in Patient 8 was performed at the Department of Medical Genetics, University of Oslo. Genomic DNA from the patient and her parents was extracted from peripheral blood. Sequencing was performed as described by McKenna *et al*. (33) and annotation was done using Annovar (http://wannovar.wglab.org) (46). Downstream filtering and analysis were done with Filtus (47) on the variants within coding regions and intron/exon boundaries. A trio-based inheritance filtering was used focusing on *de novo*, recessive or X-linked variants.

Variants were classified according to the American College of Medical Genetics (ACMG) (48) and the Association for Clinical Genomic Science (ACGS) (49) guidelines for variant interpretation.

### Protein modelling

Modelling of the DEPDC5 missense variants p.Thr337Arg and p.Arg806Cys was carried out using the FoldX modelling suite (50), which also provides quantitative values for ΔΔ*G*, the thermodynamic impact of variants on protein stability. Variants were also assessed using the Missense3D prediction tool (http://missense3d.bc.ic.ac.uk/missense3d), which assesses the structural impact of missense variants on protein structure by a number of objective criteria (51). All structures were visualized in PyMOL (PyMOL Molecular Graphics System, Version 2.0, Schrödinger LLC; New York, NY, USA).

### Pathological examination

A skin sample from Patient 4 was used for immunohistochemistry. Post mortem examinations were either not suggested, or declined by the families.

### Immunohistochemistry

The phospho-S6 ribosomal protein (PS6; clone Ser235/236, Cell Signalling #2211) and phosphor-4E-BP1 (P4EBP-1; clone Thr37/46, 236B4, Cell Signalling #2855) antibodies were used in the dilution of 1:50 and 1:200, respectively. The HIER 30 ER2 antigen retrieval method was used and Leica Bond-Max automated immunohistochemistry for antigen detection was done as per the manufacturer’s protocol. Briefly, Leica bond detection kit (Leica #DS9800) for both antibodies was used containing the post primary (secondary) antibody—anti-mouse IgG (<10 μg/ml) in 10% (v/v) animal serum in tris-buffered saline/0.09% ProClin™ 950, DAB substrate chromogen and haematoxylin counterstain. A general control tissue micro-array was used for controls, which included a normal skin sample from excision of polydactyly in a newborn (presumed negative control). In addition, the controls for PS6 included a brain sample from FCD in a 5-year-old (positive control).

## Funding

The work was supported by the Epilepsy Society. This work was partly carried out at NIHR University College London Hospitals Biomedical Research Centre, which receives a proportion of funding from the UK Department of Health’s NIHR Biomedical Research Centres funding scheme. The 100 000 Genomes Project is managed by Genomics England Limited (a wholly owned company of the Department of Health and Social Care UK). The 100 000 Genomes Project is funded by the NIHR and NHS England. The Wellcome Trust, Cancer Research UK and the Medical Research Council have also funded research infrastructure. The 100 000 Genomes Project uses data provided by individuals and collected by the NHS as part of their care and support. This work was supported by grants from the Swiss National Science Foundation (31003A_182632), the Lejeune Foundation (#1838-2019A) and the Blackswan Foundation to AR.

## Supplementary Material

Supplemental_table_1_tiered_variants_from_WGS_of_Patient_1_ddac225

Supplemental_table_2-shared_rare_variants_Patients_6_and_7_ddac225

Supplemental_table_3-ROH_comparison_patients_1_2_3_and_4-Revised_ddac225

Supplement_1_ddac225

Supplement_2_revised_ddac225
